# Prediction of early postoperative major cardiac events after elective orthopedic surgery: the role of B-type natriuretic peptide, the revised cardiac risk index, and ASA class

**DOI:** 10.1186/1471-2253-14-20

**Published:** 2014-03-21

**Authors:** Luigi Vetrugno, Nicola Langiano, Renato Gisonni, Alessandro Rizzardo, Paola Enrica Venchiarutti, Michele Divella, Livia Pompei, Araldo Causero, Giorgio Della Rocca

**Affiliations:** 1 Dipartimento di Scienze Mediche Sperimentali e Cliniche, Università degli Studi di Udine, P.le Kolbe 3, ZIP code: 33100 Udine, Italy; 2 Department of Anesthesia and Intensive Care Medicine, University-Hospital of Udine, P.le S. M. della Misericordia 15, ZIP code: 33100 Udine, Italy; 3 Department of Orthopedic Surgery, University-Hospital of Udine, P.le S. M. della Misericordia 15, ZIP code: 33100 Udine, Italy

**Keywords:** Brain natriuretic peptide, Orthopedic surgery, Preoperative care

## Abstract

**Background:**

The aim of this study was to evaluate pre- and post-operative brain natriuretic peptide (BNP) levels and compare the power of this test in predicting in-hospital major adverse cardiac events (MACE: atrial fibrillation, flutter, acute heart failure or non-fatal/fatal myocardial infarction) in patients undergoing elective prosthesis orthopedic surgery to that of the Revised Cardiac Risk Index (RCRI) and American Society of Anesthesiology (ASA) class, the most useful scores identified to date.

**Methods:**

The study was an observational study of consecutive patients undergoing elective prosthesis orthopedic surgery. Surgical risk was established using RCRI score and ASA class criteria. Venous blood was sampled before surgery and on postoperative day 1 for the measurement of BNP. The intraoperative data collected included details of the surgery and anesthesia and any MACE experienced up until hospital discharge.

**Results:**

MACE occurred in 14 of the 227 patients treated (6.2%). Age was statistical associated with MACE (p < 0.004). Preoperative BNP levels were higher (p < 0.0007) in patients who experienced MACE than in event-free patients (median values: 92 and 35 pg/mL, respectively). Postoperative BNP levels were also greater (p < 0.0001) in patients sustaining MACE than in event-free patients (median values: 165 and 45 pg/mL, respectively). ROC curve analysis demonstrated that for a cut-off point ≥ 39 pg/mL, the area under the curve for preoperative BNP was equal to 0.77, while a postoperative BNP cut-off point ≥ 69 pg/mL gave an AUC of 0.82.

**Conclusions:**

Both pre- and post-operative BNP concentrations are predictors of MACE in patients undergoing elective prosthesis orthopedic surgery.

## Background

The cardiopulmonary exercise test (CPET) is generally considered as the “gold standard” in the assessment of exercise capacity [1,2]. However, patients undergoing elective major orthopedic surgery associated with severe postoperative morbidity (including cardiac ischemic events) may not able to undergo this or other subjective measures of cardiorespiratory reserve, such as metabolic equivalency tests (METs) [3,4], since mobility in these patients may be limited [5]. Moreover, in patients at increased risk of postoperative morbidity, non-invasive stress testing (presumed to simulate the adrenergic stress of surgery and the perioperative period) is expensive and has been shown to have a low predictive positive value (i.e., it has a high false positive rate), which might cause more harm than good [6,7]. To overcome this limitation and to identify patients at increased risk of postoperative morbidity, including cardiac events, several surgical scoring systems have been developed, such as the Revised Cardiac Risk Index (RCRI) [8], the modified RCRI [9,10], and the Postoperative Morbidity Survey [11]. But once again, the ability of such tests to detect postoperative morbidity is only modest [9].

Brain natriuretic peptide (BNP) is a 32-amino acid peptide containing a 17-amino acid ring structure common to all natriuretic peptides. Measurement of plasma levels of BNP or its N-terminal fragment (NT-pro-BNP) is being increasingly used in the perioperative setting [12]. Recently, a number of studies have examined the hypothesis that elevated preoperative plasma concentrations of natriuretic peptides can be used to identify patients at risk of adverse cardiac events [13,14].

The aim of this study was to evaluate the power of pre- and post-operative BNP levels to predict in-hospital major cardiac events (MACE: atrial fibrillation or flutter, acute heart failure, or non-fatal/fatal myocardial infarction) in patients undergoing elective prosthesis orthopedic surgery, and to compare it to that of the RCRI and American Society of Anesthesiology (ASA) class – the most useful scores identified to date.

## Methods

This study was approved by the Ethics Committee of the University-Hospital of Udine “S. Maria della Misericordia” and all patients gave their written informed consent for participation. The study was an observational cross study of consecutive patients undergoing elective prosthesis orthopedic surgery. Elective prosthesis orthopedic surgery was defined as first implant of the hip or knee replacement. Patients were included if they were >50 years of age and undergoing spinal anesthesia. Exclusion criteria were: lack of consent, atrial fibrillation, a recent history (within 6 months) of unstable coronary syndrome, or decompensate heart failure. Since severe aortic valve stenosis and impaired renal function are associated with increased serum levels of natriuretic peptides, patients with these preoperative diagnoses were also excluded. Before surgery, patients underwent routine clinical evaluation, including the taking of a detailed medical history, a physical evaluation, routine laboratory tests, 12-lead electrocardiography and chest radiography. Clinically stable patients with coronary artery disease proceeded to surgery without any further non-invasive testing [15]. Surgical risk in each patient was established using the criteria of the ASA [16] (the criteria of this classification are well-known and patients are classified as ASA I, II, III, IV or V) and the RCRI as defined by Lee et al. [8]. Patients underwent venous blood sampling in the morning of the pre-anesthesia visit (pre-hospitalization). However, in cases in which this was not possible, blood samples for assessing pre-operative BNP were drawn on the same day as surgery. Postoperative BNP was sampled in the morning of the first day after surgery. Blood was collected in EDTA and lithium heparin vacutainer tubes, and immediately send to the laboratory for analysis (by means of a specialized transport service for biological materials) where BNP concentrations were assayed using the Bayer ADVIA Centaur™.

The results of these tests were not known in real time and investigators and patients were blind to the test results. Anesthesia was conducted by consultant anesthetists. Intrathecal injections were performed in the pre-operating room. Patients received either hyperbaric bupivacaine 5 mg/mL (2 mL) (Angelini-A.C.R.A.F, Rome/Italy) or isobaric levobupivacaine 5 mg/mL (Abbot S.r.l., Campoverde di Aprilia, Latina, Italy), according to the anesthetist’s choice.

Perioperative data collection included details of the surgery and anesthesia and any major adverse cardiac events (MACE: new onset of atrial fibrillation, flutter, acute heart failure or non-fatal/fatal myocardial infarction) occurring prior to hospital discharge. In particular, postoperative acute myocardial infarction (AMI) was defined for Troponin I (cTnI) concentrations ≥0.12 ng/mL (3 times the 99th percentile upper reference limit (URL) in patients presenting specific features (clinical or ECG signs), in accordance with the definition of myocardial infarction used in previous clinical investigations [17].

Pre- and post-operative creatinine, pre- and post-operative hemoglobin (Hb), the lowest Hb value, the number of transfusion units, and the overall surgical time were recorded. Postoperative pain management consisted of oral oxycodone 10 mg (BARD Pharmaceutics Ltd., Cambridge, United Kingdom) and was administered before and 12 hours after hip surgery, together with 10–15 mg/kg i.v. acetaminophen (Fresenius Kabi S.r.l, Verona, Italy), which was given every 6 hours. After knee surgery, continuous femoral nerve blockade was obtained using levobupivacaine 4 mg/hour, delivered through a PlexoLong Nanoline Tuohy cannula (PAJUNK GmbH, 18G × 50 mm Medizintechnologie, Geisingen, Germany); this perineuronal continuous infusion was used instead of 10 mg oral oxycodone.

For the entire sample, as well as for the sample stratified for the presence or absence of MACE, median values (plus IQR) were calculated for: age, gender, body mass index (BMI), pre- and post-operative creatinine, pre- and post-operative hemoglobin and its lowest intra-operative value, transfusion units and surgical time; while percentage distributions were calculated for: RCRI score, ASA class, length of stay (LOS), the presence of coexisting diseases, and drugs. The percentage distributions for the continuous variables in patients with MACE and in patients without MACE were tested using Fisher’s exact *t* test or the chi squared test. Pre- and postoperative BNP and the other quantitative variables were investigated using the non parametric Mann–Whitney *U* test for independent samples for the two group considered. Receiver operating characteristic (ROC) curves were plotted and the area under the curve (AUC) estimated to establish a cut-off of the pre- and post-operative BNP values with appropriate sensitivity and specificity to prevent MACE. The optimum cut-off point was determined as the value corresponding to the greatest accuracy (i.e. highest summed value for sensitivity plus specificity values, with sensitivity and specificity weighted equally). ROC curves were also calculated to establish the AUC for RCRI and ASA class. Univariate logistic regression analyses were used to study how patient variables, the presence of coexisting disease, and medication history influenced the occurrence of MACE. Multiple logistic regression analysis was used to test the predictive value of elevated BNP levels and other selected parameters. Factors with a p-value <0.25 in univariate analysis were entered into a multivariate model. Finally, as described by Rodseth et al., patients were categorized according to their RCRI group (0 = low risk; 1 or 2 = intermediate risk; 3, 4 or 5 = high risk) and then reclassified according to their preoperative BNP (above or below the cut-off) [18].

Our own pilot data [19] on vascular surgery patients suggest that the incidence of AMI is around 11%, with these patients presenting a mean preoperative BNP level of 209 pg/mL (interquartile range, IQR: 84–346) compared to 74 pg/mL (IQR: 28–142) in those who did not. Thus, we calculate that a sample size of 200 patients completing the study for preoperative BNP and 206 patients for postoperative BNP would allow us to detect a 30 pg/mL difference in BNP levels between patients who did and those who did not experience acute myocardial infarction, with 90% statistical power at the 5% significance level. The software SAS 9.3 for Windows (SAS Institute Inc., Cary, North Carolina, USA) was used for all statistical analyses.

## Results

**Figure 1:**
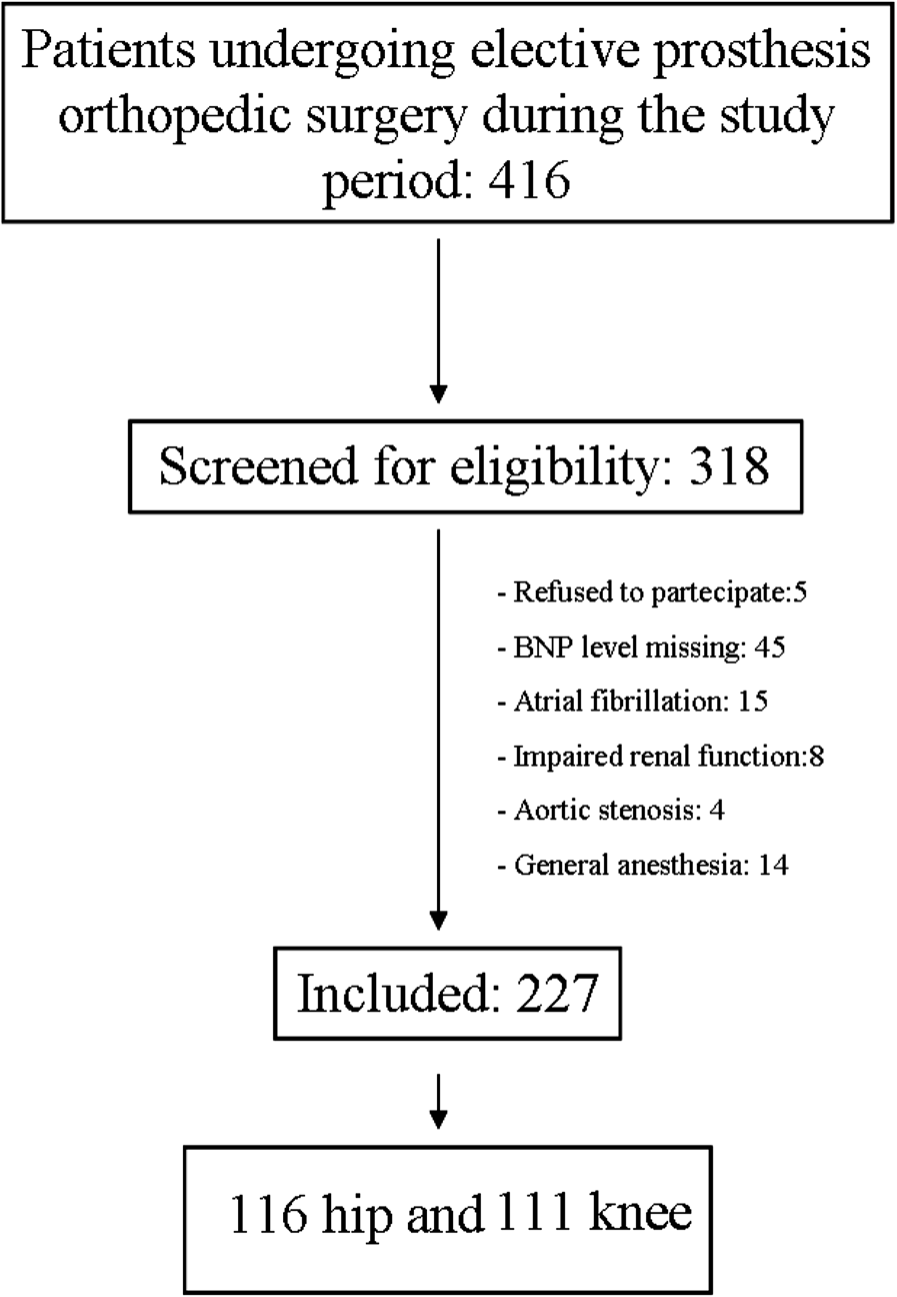
Diagram of the patient flow through the study.

Four-hundred-sixteen patients underwent elective prosthesis orthopedic surgery during the study period (July 2010 to June 2012). Of these, 318 patients were screened for eligibility, of which 227 fulfilled the inclusion criteria and were prospectively enrolled onto the study: 116 patients underwent hip replacement surgery and 111 knee replacement surgery under spinal anesthesia, with 14 requiring rescue (Figure 1). The demographic characteristics, ASA class, RCRI, coexisting diseases, and medication history for this cohort are shown in Table 1. Postoperative MACE occurred in 14 of the 227 patients treated: 7 cases of acute (non fatal) AMI; 5 cases of AF, and 2 cases of bradycardia with new onset atrio-ventricular block. Of the demographic variables, only age was significantly associated with MACE. Previous angina and previous myocardial infarction showed only marginal association (Table 1), as was also the case for lowest intra-operative hemoglobin level (Table 2). Preoperative BNP levels were higher (p < 0.0007) in patients who experienced MACE (median value 92 pg/mL, IQR: 45–143) than in event-free patients (median value 35 pg/mL, IQR: 19–74). Postoperative BNP levels were also greater (p < 0.0001) in patients sustaining MACE (median 165 pg/mL, IQR: 71–214) than in event-free patients (median value 45 pg/mL, IQR: 27–86; Figure 2 and Table 2). Analysis of the ROC curve (Figure 3) demonstrates that for a cut-off point ≥ 39 pg/mL, preoperative BNP showed a sensitivity of 93% and specificity of 56% in predicting events, with an AUC of 0.77 (95% confidence interval (CI): 0.66 – 0.87). A postoperative BNP cut-off point ≥ 69 pg/mL showed a sensitivity of 86% and specificity of 68%, with an AUC of 0.82 (95% CI: 0.70 – 0.93). Patients who experienced MACE (n = 14) remained in hospital for a median of 10 days (IQR: 8–14) following their operation, compared with 8 (6–9) days in those who were event-free (p < 0.0016). Of the 14 patients experiencing MACE, 10 were categorized as ASA class II (71%), and 4 as class III (29%); statistical analysis confirm a significant difference in the distribution between the two groups (p < 0.05). When patients were stratified by RCRI score, MACE events occurred more frequently in patients with low or intermediated RCRI (p < 0.09). ROC curve analysis for assessing the capacity of ASA and RCRI to predict MACE gave AUC of 0.59 (95% CI: 0.47 – 0.71) and 0.63 (95% CI: 0.48 – 0.77), respectively. Univariate regression analysis showed that age (p < 0.001) and lowest intra-operative hemoglobin level (p < 0.047) were statistically associated with MACE, as were preoperative BNP concentrations ≥ 39 pg/mL (p < 0.0007) and postoperative BNP concentrations ≥ 69 pg/mL (p < 0.0001). Multivariate regression analysis showed that the preoperative variables with p values <0.25 were statistically different from age: odd-ratio (OR) of 1.114 (95% CI: 1.002 – 1.238); the same was true for preoperative BNP concentrations ≥ 39 pg/mL: OR = 9.007 (95% CI: 1.051 – 77.191). In the postoperative period, only postoperative BNP concentrations ≥ 69 pg/mL showed a significant difference: OR = 6.371 (95% CI: 1.129 – 35.94).

**Table 1:** Patients population with and without MACE

	**Entire sample**	**MACE**	**NO MACE**	***p value***
	**n = 227**	**(n = 14)**	**(n = 213)**	
**Demographic characteristics**				
Age; yrs (median, IQR)	71 (66–76)	78 (74–82)	71 (66–75)	0.0004^1^
Male; n (%)	90 (40)	3 (21)	87 (41)	0.15^2^
Female; n (%)	137 (60)	11 (79)	126 (59)	
BMI; Kg/m^2^ (median, IQR)	28 (25–31)	27 (25–29)	28 (25–31)	0.44^1^
Obesity; n (%)	98 (43)	5 (36)	93 (44)	0.56^2^
**ASA class; n (%)**				
Class I	10 (4)	0 (0.00)	10 (4)	0.05^3^
Class II	184 (81)	10 (71)	174 (82)	
Class III	33 (15)	4 (29)	29 (14)	
**RCRI; n (%)**				
0	165 (72.7)	7 (50)	158 (74.1)	0.09^3^
1	44 (19.4)	4 (28.6)	40 (18.8)	
2	10 (4.4)	2 (14.3)	8 (3.8)	
3	7 (3.1)	1 (7.1)	6 (2.9)	
4	1 (0.4)	0 (0.00)	1 (0.4)	
**Coexisting diseases; n (%)**				
Hypertension	169 (74)	12 (86)	157 (74)	0.17^3^
Diabetes	40 (18)	3 (21)	37 (17)	0.72^3^
Smokers	40 (18)	1 (7)	39 (18)	0.47^3^
COPD	12 (5)	0 (0.00)	12 (5.6)	1.00^3^
Previous angina	18 (8)	3 (21)	15 (7)	0.09^3^
Previous AMI	17 (7)	3 (21)	14 (6.5)	0.07^3^
Previous AF	15 (6.6)	2 (14)	13 (6.1)	0.23^3^
**Medication history; n (%)**				
ACE inhibitor	43 (19)	5 (36)	38 (18)	0.15^3^
Aspirin	43 (19)	4 (29)	39 (18)	0.30^3^
Beta-blockers	64 (28)	6 (43)	58 (28)	0.23^3^
Ca antagonists	25 (11)	1 (7)	24 (11)	1.00^3^
Diuretics	54 (24)	4 (29)	50 (23)	0.44^3^
AR antagonist	67 (30)	3 (21)	64 (30)	0.84^3^
Statins	45 (20)	2 (15)	43 (20)	0.74^3^

P values indicate the significance of the association to MACE.

NOTE: The data are presented with median and interquartile range (IQR) or percentage (%).

Legend: MACE (major adverse cardiac event), ASA (American Society of Anesthesiology), BMI (Body mass index), RCRI (revised cardiac risk index), COPD (chronic obstructive pulmonary disease), AMI (acute myocardial infarction), AF (atrial fibrillation), ACE (angiotensin-converting enzyme), AR (angiotensin receptor).

^1^*P* value derived using Mann–Whitney U test. ^2^*P* value derived using Chi-squared test for quantitative variables. ^3^*P* value derived *Fisher’s exact test* for qualitative variables.

**Table 2:** Pre- and postoperative blood tests, transfusion units, surgical time and LOS

**Laboratory tests and intra-operative data**	**Entire sample**	**MACE**	**NO MACE**	***p value***
**(Median values, plus IQR)**	**n = 227**	**(n = 14)**	**(n = 213)**	
Preop. Creat (mg/dL)	0.93 (0.80-1.07)	0.95 (0.86-1.10)	0.92 (0.80-1.06)	0.49
Postop. Creat (mg/dL)	0.85 (0.72-0.98)	0.87 (0.74-0.93)	0.85 (0.72-0.99)	0.99
Preop. Hb (mg/dL)	13.5 (12.8-14.5)	13.15 (12.4-15.1)	13.50 (12.80-14.40)	0.99
Postop. Hb (mg/dL)	9.6 (8.6-11.3)	8.75 (7.4-10)	9.60 (8.60-11.30)	0.13
Lowest intraop. Hb (mg/dL)	11.1 (9.9-12.4)	10.05 (8.6-11.5)	11.15 (9.90-12.40)	0.08
BNP preop. (pg/mL)	36 (20–77)	92 (45–143)	35 (19–74)	0.0007
BNP postop. (pg/mL)	48 (28–97)	165 (71–214)	45 (27–86)	0.0001
Transfusion	0 (0–0)	0 (0–2)	0 (0–0)	0.18
Surgical time (min)	100 (75–120)	107 (85–150)	100 (75–120)	0.17
LOS (days)	8 (6–9)	10 (8–14)	8 (6–9)	0.0016

P values indicate the significance of the association to MACE.

Legend: Preop. (preoperative), Postop. (postoperative), Intraop. (intraoperative), Creat (creatinine), MACE (major adverse cardiac event), Hemoglobin (Hb), BNP (Brain Natriuretic Peptide), LOS (Length of stay).

*P* value derived using Mann–Whitney U test.

**Figure 2:**
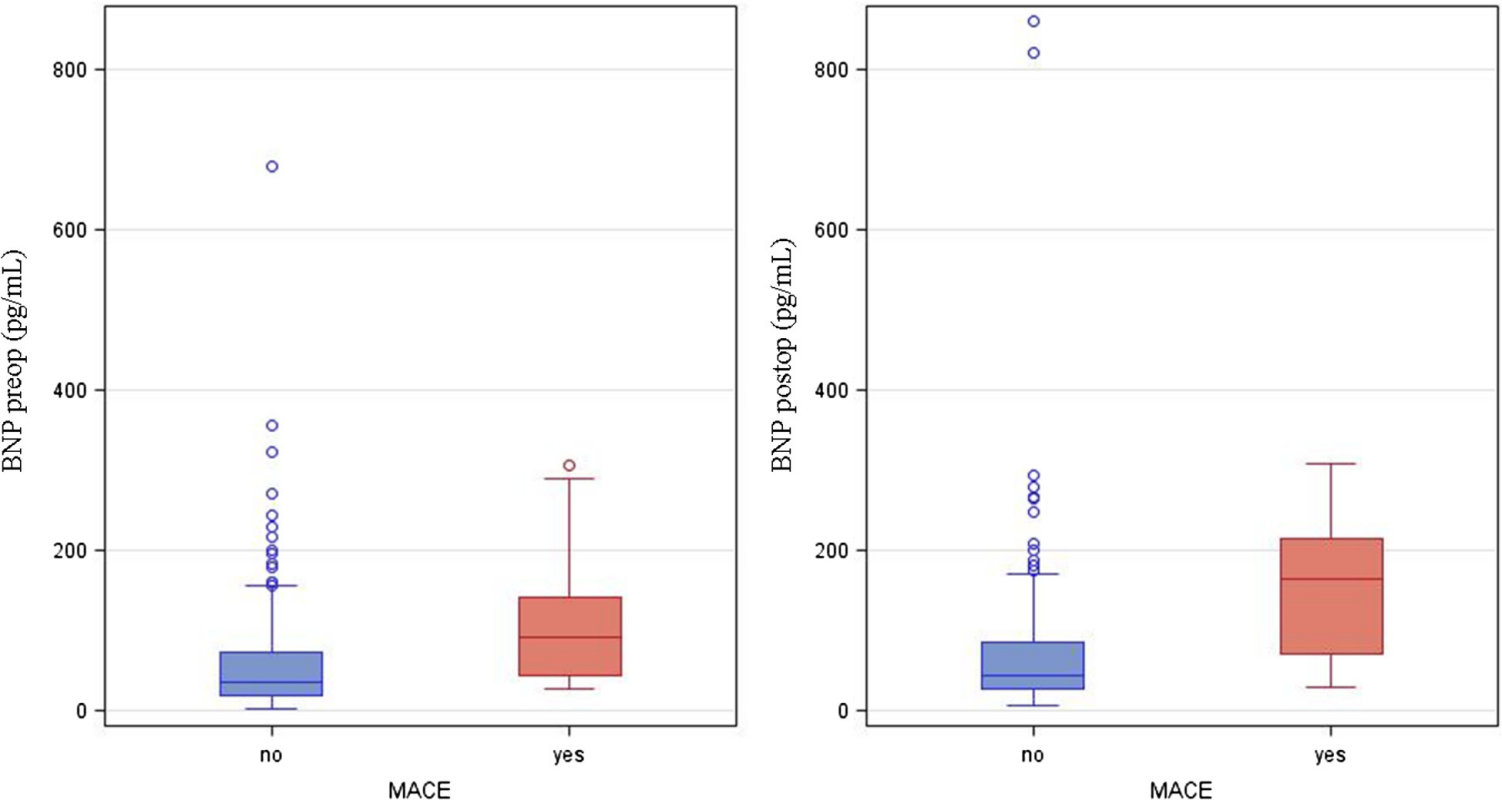
**Box plots of preoperative (Preop.) and postoperative (Postop.) BNP levels in the 14 patients with MACE (red bars) compared with the event-free patients (blue bars).** P <0.0007 BNP Preop. vs P <0.0001 Postop.

**Figure 3:**
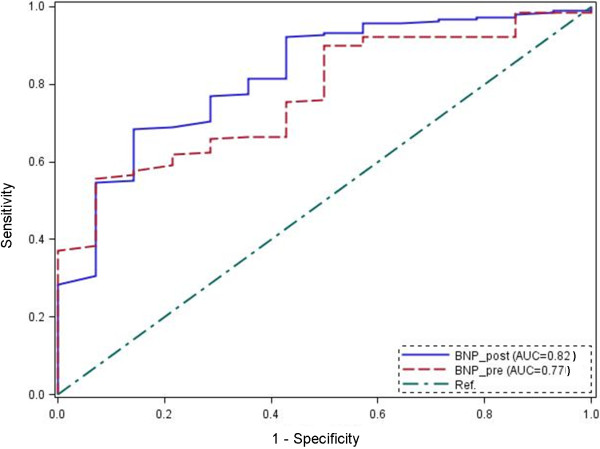
**Receiver operating characteristic curve for preoperative (Preop.) and postoperative (Postop.) BNP levels and prediction of in-hospital MACE (major cardiac evets).** AUC = area under the curve, Ref. = reference line.

All 227 patients were classified into 3 risk groups according to their RCRI scores [18] (low 0, intermediate 1–2, high 3-4-5). A reclassification was also performed on the basis of the preoperative BNP levels. If levels fell below 39 pg/mL, patients were moved down by 1 risk category, and if the levels rose above 39 pg/mL, patients were moved up 1 risk category; Table 3 shows the results of the reclassification process. Of the patients originally classified into the low risk group, 40.6% became reclassified as intermediate when considering preoperative BNP. In patients classified into the intermediate risk group, 37% became reclassified into the low and 63% into the high risk group. Re-stratifying according to preoperative BNP concentrations resulted in a statistically significant improvement in discrimination (Table 3).

**Table 3:** Risk reclassification with preoperative BNP cutoff ≥ 39 pg/dL

**RCRI risk category**	**Entire sample**	**MACE**	**NO-MACE**	**Reclassification RCRI**	**MACE**	**NO-MACE**
Low risk	165 (72.7)	7 (50)	158 (74.1)	118 (52)	1 (7.1)	117 (55)
Intermediate risk	54 (23.8)	6 (42.9)	48 (22.6)	67 (29.5)	6 (42.9)	61 (28.6)
High risk	8 (3.5)	1 (7.1)	7 (3.3)	42 (18.5)	7 (50)	35 (16.4)

Legend: RCRI (revised cardiac risk index), MACE (major adverse cardiac event).

In the time period prior to hospital discharge, 5 of the cohort patients experienced acute respiratory failure (3 pneumonia and 2 acute pulmonary embolisms), four developed acute renal failure, 6 developed an infection (2 cases of early prosthesis infection and 4 cases of urinary tract infection), and 5 patients developed neurological dysfunctions. The results of the Chi Squared test show that there is no statistically significant association between the dichotomized preoperative BNP level and the occurrence of other (non-cardiac) complications (p = 0.8782). Logistic regressions show that: i) preoperative BNP ≥ 39 pg/dL does not increase the risk of further complications (OR = 0.919, 95% CI: 0.312 – 2.710); and ii) an increase in preoperative BNP is not associated with a statistically significant higher risk of further complications (OR = 0.996 95% CI: 0.991 – 1.001).

## Discussion

The main findings of this study are two-fold: first, pre- and post-operative BNP levels are predictors of perioperative MACE in patients undergoing prosthesis elective orthopedic surgery under spinal anesthesia; second, RCRI and ASA appear to be inadequate predictors of postoperative cardiac events in this form of elective surgery. The data also confirm that in-hospital non-cardiac morbidity (acute respiratory failure, acute renal failure, infections, and neurological deficit) is more common than MACE (8.8% versus 6.2%), as previously reported [10]. The use of objective measures of cardiopulmonary reserve, including cardiopulmonary exercise testing, has been reported in the orthopedic literature, but such methods are often deemed to be impractical. For example, in a study of the patients with end-stage arthritis of the hip and knee, only 60% were able to undertake conventional bicycle ergometry [20]. Current international guidelines recommend that the subjective assessment of functional capacity (METs) forms one of the three key criteria in the decision-making process underlying risk stratification prior to non-cardiac surgery [15]. A functional capacity of <4 METs forms one of the threshold values that direct a patient down the high risk pathway of risk stratification. The intrinsic limitation of assessing exercise capacity in orthopedic patients plus the cost and expertise required to carry out preoperative tests both point to the need for an alternative objective testing method for assessing perioperative risk.

BNP is a 32-amino acid peptide containing a 17-amino acid ring structure common to all natriuretic peptides [21,22]. BNP levels in adults strongly correlate with the New York Heart Association (NYHA) functional classification of patients with heart failure and have been shown to act as an independent predictor of adverse outcome [23]. N-terminal pro-brain natriuretic peptide (NT-pro-BNP), an inactive by-product resulting from the synthesis of BNP, is another member of the natriuretic peptide family [21]. Measurement of either plasma BNP or NT-pro-BNP levels is being increasingly used in the perioperative setting [9,12,14]. Yeh and colleagues investigated patients undergoing non-cardiac surgery and were the first to describe the capacity of NT-pro-BNP to predict the composite end-point of postoperative cardiac complications [24]. Breidthardt et al. studied patients undergoing “unselected” orthopedic surgery and demonstrated that preoperative BNP levels can predict short-term and long-term postoperative cardiac events, with ROC analysis giving an AUC of 0.86 for preoperative BNP [25]. Moreover, in a recent meta-analysis of pre- and post-operative BNP values, Rodseth et al. found that patients with elevated postoperative BNP concentrations were at increased risk of AMI, cardiac failure and mortality at 30 days and even 180 days after surgery [26]. The usefulness of preoperative BNP measurements as a predictor of perioperative cardiac events was also reported by Cuthbertson and co-workers, who identified a cut-off of preoperative BNP > 40 pg/mL to be associated with a 5-fold increase in the risk of developing either postoperative cTnI elevation > 0.32 ng/mL or new ECG abnormalities [27]. Using the optimum cut-off point, we identified a very similar preoperative BNP threshold of ≥ 39 pg/mL, which was associated with a 9-fold increase in MACE. We also found that a postoperative BNP threshold of > 69 pg/mL corresponded to a 6-fold increase in MACE risk. Univariate and multivariate analysis of the data gathered consistently identified the demographic factor age to be an independent predictor of MACE events, with an increase in the OR of 1.114 per year. Our study is the first to examine the power of both pre- and postoperative BNP levels in predicting MACE in a selected population of patients undergoing elective orthopedic prosthetic surgery. However, four other studies have reported similar findings in the orthopedic setting. Chong CP and colleagues reported their observations in relation to NT-pro-BNP after emergency orthopedic-geriatric surgery, with rates of in-hospital cardiac complications and AMI of 26% and 13.4%, respectively [12]. It should be noted that the average age of their population was 79.9 ± 9.6 years. Oscarsson A. and colleagues investigated the utility of BNP to predict perioperative cardiac events in high-risk patients undergoing major non-cardiac surgery [28]. Their patient population fell into ASA class III or IV categories, and 49% of which experienced a perioperative adverse cardiac event. Villacorta and co-workers investigated preoperative BNP in patients undergoing orthopedic elective surgery where 8% of patients experienced a cardiac event [14]. They similarly found ASA classification to be a poor predictor of cardiac events. Finally, Montagnana and colleagues enrolled 37 patients undergoing major elective orthopedic surgery and found that NT-pro-BNP levels were significantly increased 72 hours postoperatively compared to preoperative levels and assumed it to be a consequence of fluid overload [29]. A recent meta-analysis raised concerns about the usefulness of the RCRI and the performance of non invasive tests and imaging studies, as they revealed them to have a low positive predictive value (PPV) [9]. Our study confirms this finding by revealing an AUC of just 0.63 (95% CI: 0.48 – 0.77) in the ROC curve analysis of RCRI, in accordance with the results of recent publications [10,28]. The low predictive power of RCRI indicated by these studies supports the need for other objective measures to be explored. However, if RCRI is used, then a reclassification of patients according to pre-operative BNP levels should be performed, as discussed by Rodseth and colleagues, in order to improve its power [18].

In summary, the present study indicates the assessment of BNP to be more reliable than ASA and RCRI in predicting adverse cardiovascular outcomes following non-cardiac surgery. We can therefore propose the following: i) the preoperative BNP cut-off value provide a more suitable tool for the stratification of preoperative risk, and ii) increased postoperative BNP levels could be used to signal the need for hemodynamic optimization in the appropriate setting and depending on the resources available.

Several limitations of the present study need to be acknowledged: first of all, the number of clinical events is relatively small and cardiovascular morbidity – specifically, cardiac ischemia – was a relatively infrequent morbidity domain (3%). In our population, the clinical relevance of incidental rises in postoperative troponin is uncertain as is the data regarding ejection fraction. We also exclude patients with impaired renal function and this could represent another limitation of the study because in clinical practice renal failure is quite common. Last but not least, our definition of MACE, which included atrial fibrillation and flutter, was very broad.

## Conclusion

In conclusion, our results show both pre- and post-operative BNP to be predictors of MACE events. BNP assessment should therefore be performed to help the clinician to stratify cardiac risk and improve the evaluation of preoperative cardiovascular status in patients undergoing elective prosthesis orthopedic surgery. In our setting, BNP did not predict all causes of morbidity well, as was to be expected since it cannot capture risk factors for non-cardiac causes of morbidity or other perioperative complications.

## Competing interests

The authors declare that they have no competing interests.

## Authors’ contributions

LV and GDR planned the study, were responsible for its design and coordination and drafted the manuscript. AR, NL, DM, RG and PEV participated in the study design and collected patient data. AC, RG and LP also help to draft the manuscript. All authors read and approved the final manuscript.

## Pre-publication history

The pre-publication history for this paper can be accessed here:

http://www.biomedcentral.com/1471-2253/14/20/prepub
